# A qualitative study to understand sociocultural beliefs around perinatal and neonatal health in rural areas of Mohali, Punjab, India

**DOI:** 10.3389/fgwh.2023.1147762

**Published:** 2023-11-30

**Authors:** Alka Ahuja, Mona Duggal, Jane Y. Liu, Preetika Sharma, Darshan Hosapatna Basavarajappa, Rashmi Bagga, Alison M. El Ayadi, Ankita Kankaria, Vijay Kumar, Pushpendra Singh, Nadia G. Diamond-Smith

**Affiliations:** ^1^ Department of Obstetrics and Gynecology, Post Graduate Institute of Medical Education and Research (PGIMER), Chandigarh, India; ^2^ Advanced Eye Centre, Post-Graduate Institute of Medical Education and Research, Chandigarh, India; ^3^ School of Public Health, University of California Berkeley, Berkeley, CA, United States; ^4^ Department of Obstetrics, Gynecology and Reproductive Sciences, University of California San Francisco, San Francisco, CA, United States; ^5^ Department of Community and Family Medicine, All India Institute of Medical Sciences, Bathinda, India; ^6^ Survival for Women and Children Foundation, Panchkula, India; ^7^ Department of Computer Science and Engineering, Indraprastha Institute of Information Technology, Delhi, India; ^8^ Department of Epidemiology and Biostatistics, University of California San Francisco, San Francisco, CA, United States

**Keywords:** sociocultural, beliefs, practices, pregnancy, postpartum, child health

## Abstract

**Introduction:**

Globally, 600,000 mothers (15-49 years) die every year due to pregnancy and childbirth-related complications. Wide variations are seen in cultural practices and beliefs surrounding this period of a woman's life. The present study explores the cultural beliefs and practices of women and families during pregnancy and the postnatal period in order to understand what behavioral management strategies are required to improve maternal and infant outcomes during pregnancy and the postpartum period.

**Methods:**

The study was conducted in a rural area of Punjab, from December 2019 to March 2021. A total of 20 women (up to 3 months postpartum, age >18 years, were interviewed.

**Results:**

In general, women described eating varied and fairly healthy diets during pregnancy, especially nutritious warm food, following traditional practices. Other cultural practices included restrictions on movement and mobile phone use and the use of unsafe home remedies to promote infant safety and wellbeing, such as using gripe water, applying black pencil to the baby's eyes, and feeding the baby honey. A few were not inclined to engage with these and other cultural expectations, preferring instead to follow contemporary practices influenced by social media. These practices included being accompanied by a family member during delivery, celebrating the baby's birth regardless of sex, and early bathing post-delivery.

**Discussion:**

It can be concluded that while many traditional practices are still followed in India, there are new beliefs and behaviors arising from an intersection between culture and technology. Developing strategies that acknowledge older beliefs and modern approaches is essential to promoting better antenatal and postpartum care.

## 1. Introduction

Globally, approximately nearly 600,000 mothers in the age group of 15–49 years die every year from pregnancy and childbirth-related complications (1). The major reasons behind this are complications arising from pregnancy and childbirth. The majority (99%) of these maternal deaths occur in developing countries (1). Almost 50% of postnatal deaths occur within the first 24 h of birth and 66% occur during the first week of the postnatal period. The major causes of maternal mortality include hemorrhage, pregnancy-induced hypertension (PIH), puerperal sepsis, ruptured uterus, anemia, and obstructed labor, all of which can be compounded by more distal factors such as malnutrition and poverty (2).

The infant mortality rate in India decreased from 57 per 1,000 live births in 2005–2006 (National Family Health Survey 3) to 41 in 2015–2016 (National Family Health Survey 4) and to 35 in 2019–2021 (National Family Health Survey 5) (3, 4). However, this figure is still high. The distribution of these gains is uneven across states and between urban and rural locations (5). The infant mortality rate in Punjab in NFHS-4 is estimated at 29 deaths before the age of one year per 1,000 live births, down from the NFHS-3 estimate of 42, and the NFHS-2 estimate of 57 (6). Only 30.7% of children under the age of 3 years were breastfed within 1 h of birth and 53.0% of children were exclusively breastfed up to 6 months, which contributed to poor infant outcomes (6).

As per a nationally representative survey (NFHS 4, 2015–2016) from India, Punjab state had only 30.7% of women receiving complete antenatal care (women aged 15–49 with a live birth in a given time period receiving antenatal care four or more times) (7). Although the percentage of women who had at least one postpartum health check in the 2 months after delivery increased from 53.1% (NFHS 3) to 87.2% (NFHS 4), only 40.7% of children received a health check from a doctor/nurse/LHV/ANM/ midwife/other health personnel within 2 days of birth (6).

Access-related factors can influence care-seeking practices and behaviors in pregnancy and postpartum, such as the logistical challenges of traveling to the health facility with a young baby or women living far from their health facility (rural or tribal area), particularly in India (8).

Besides these structural factors, cultural practices also influence women's behavior and outcomes during the pregnancy and postpartum period. There are wide variations in cultural practices and beliefs surrounding pregnancy and the postpartum period globally, which differ between countries.

While there has been some research on these cultural practices in pregnancy and postpartum in India (9, 10), much of the literature is from over a decade ago and it mostly focuses on influences on maternal diet pattern and breastfeeding practices. Much less research has looked at other sociocultural practices and how they are changing (or not) in an ever-modernizing world (9). Understanding these practices related to pregnancy and the postpartum period and planning for maternal health care education accordingly can help to improve morbidity and mortality indicators of both the mothers and their babies.

Traditional practices regarding dietary modification in pregnancy and the postpartum period in India are well documented, and pregnant women often follow the advice of their mothers-in-law (10–12). Studies have found that pregnancy diet is determined by beliefs that pregnancy contributes to body heat (13, 14) and eating “hot” food will add to this. As such, pregnant women are encouraged to avoid harmful “hot” foods (meat, eggs, and *ghee*) and eat “cold” foods (green leafy vegetables, milk) so as to avoid a miscarriage (10, 13). The opposite is recommended postpartum, where “hot” foods are encouraged and “cold” foods are avoided (11).

This is because after delivery, the woman's body has lost blood, fluids, and body heat, which is believed to put her in a “cold state”. To balance this lower body temperature, it is believed that post-partum women should be eating “hot” foods. Reducing food consumption or “eating down” during pregnancy is also practiced due to a belief that excessive consumption will result in weight gain and hence cause difficulty in delivery and fear of delivering a large baby (10, 15). While some studies have found that women do not believe reducing consumption will harm the fetus, this practice is more prevalent in under-resourced areas (10).

While significant progress has been made to improve infant feeding practices in recent years, studies still show that deep-rooted beliefs persist in feeding practices (breastfeeding and complementary feeding) in rural areas. A study conducted by Bansal et al. (2021) reported a significant association between the delay in the time of initiation of breastfeeding and cough/cold episodes was reported by (*P* value 0.039). Poor nutrition in infancy and early childhood contribute to malnutrition and may also impair cognitive and social development, resulting in poor school performance and reduced productivity in later life (16, 17).

In India, delayed breastfeeding of up to 3 days is attributed to beliefs that colostrum (fluid produced before breast milk) contains indigestible substances and will harm the newborn (10, 17, 18). Several studies have found that pre-lacteal feeding (foods such as honey, *ghee, ghutti, (*digestive medicine given to infants*)* and *jaggery* (a type of solid dark sugar made in India from sugar cane) given before breastfeeding is initiated) still exists in some rural areas of India rural and where lower levels of education and industrial development are attained (10, 19, 20). Furthermore, a delay in complementary feeding after 6 months of birth (introducing other foods when breast milk alone is no longer sufficient) was common at 6 months, and most mothers fed their infants homemade complementary food rather than commercial food (18, 19).

Common postpartum practices that persist in Indian culture are based on the belief that they benefit the health of the mother and infant. Most of the current literature on this topic focuses on cultural practices around infant care, with less documentation of practices related to postpartum women, aside from those around eating practices. The fear of harm caused to the newborns by *nazar* (evil eye) results in various practices, including placing a black dot of *kajal* (black soot mixed in butter) on the newborn's forehead and discouraging admiration of the newborn to avoid causing envy (10, 12). After delivery, the mother, along with her infant, is placed in a 40-day confinement, restricting her movements and secluding her from the outside, since it is believed to be a highly vulnerable period for the mother and infant (10, 16, 21). This period of isolation is believed to protect the new mother and infant from *nazar* and revert the “polluting” effects of giving birth (12, 16).

Understanding cultural practices in pregnancy and the postpartum period is important for designing interventions that address the full picture of the causes of certain behaviors. For example, simply providing information about the importance of early breastfeeding may not be enough without also addressing cultural beliefs around colostrum. An understanding of various traditional postnatal care practices is essential if effective behavior change strategies are to be developed and help the planners formulate effective intervention strategies and provide timely assistance to the mothers. Additionally, broadening our understanding of cultural practices in pregnancy and postpartum beyond those focused on maternal diet to really understand the wider range of influences on women's health, and how these may be shifting as India modernizes, is needed. Thus, the aim of this study was to explore the cultural beliefs and practices of women and families in one part of India (rural Punjab) during pregnancy and the postnatal period.

## 2. Materials and methods

### 2.1. Study area

The present study was conducted in Block Boothgarh, Mohali district of Punjab. Villages in Mohali are relatively densely populated and typically consist of 750–2,000 people living in approximately 150–400 households (22). According to the NFHS-4, the total fertility rate and crude birth rate in Punjab are 1.6 children per woman and 14.1 per 1,000 people per year, respectively. In total, 84.9% of women deliver in a health facility and 71.3% of women had at least four antenatal care visits during their last pregnancy (6). In rural areas of Mohali, 42.7% of women received full antenatal care [full antenatal care is at least four antenatal visits, at least one tetanus toxoid (TT) injection, and iron folic acid tablets or syrup taken for 100 or more days] (3). Only 35.8% of children under the age of 3 years were breastfed within 1 h of birth. Only 42% of children received a health check after birth from a doctor/nurse/LHV/ANM/midwife/other health personnel within 2 days of birth.

According to the “Household Survey on India's Citizen Environment & Consumer Economy” (ICE 360° survey) conducted in 2016, 88% of households in India have a mobile phone (23). Household mobile phone ownership is high in Punjab, with 90% of households and 50% of women in the Mohali district owning a mobile phone. In our target region, Punjab state, 57.2% of women own their own mobile phone.

### 2.2. Ethical consideration

This study received Ethics approval from the Institute Ethics Committee of the Postgraduate Institute of Medical Education and Research (PGIMER), Chandigarh, and the University of California, San Francisco. Permission was also received from the concerned authorities of Block Boothgarh. Potential participants were called on the telephone numbers provided by the health center and informed about the study, and if they were interested, verbal informed consent was obtained over the phone for enrolling them. Those who were interested were scheduled for an in-person interview. The Research staff (qualitative interviewer and a note-taker) visited the women at their homes, conducted the full informed consent process, obtained written confirmation of consent, and conducted the interview face-to-face.

### 2.3. CTRI registration

This research is part of the formative phase of a larger intervention trial. The trial was prospectively registered with the Clinical Trials Registry of India. (CTRI/2020/12/029800) [Registered on: 15/12/2020].

### 2.4. Recruitment

A total of 20 women were recruited through data collected from the 15 different health centers of Block Boothgarh.

### 2.5. Inclusion criteria

Women were eligible if they were primiparous, up to 3 months postpartum (post-delivery of their baby), and over the age of 18.

### 2.6. Exclusion criteria

Those not willing to participate and under 18 years of age were excluded.

### 2.7. Data collection tool

Data was collected via in-depth interviews addressing the following topics: cultural and traditional practices associated with conception, antenatal care, childbirth, postnatal care, and newborn care.

### 2.8. Data collection method

Data was collected by a qualitative researcher (a person who attempts to interpret human behavior based on the words of selected individuals). The interviews were conducted in a comfortable setting, at home (current residence with family). It was ensured that only the interviewer and the interviewee were present at the time of the interview. Interviews were audio recorded after receiving permission from the participants. The language used in the interview was either Hindi or Punjabi, based on the interviewee's preference.

### 2.9. Data collection period

The study was carried out from December 2020 to March 2021.

### 2.10. Data analysis

The average duration of the qualitative interviews was 45 min and all were audio recorded, transcribed, and translated to English for coding and analysis. A Research Team conducted a thematic analysis using the software Dedoose (24). Based on the interviews' preliminary analysis, a code guide was developed to analyze the rest of the transcribed interviews. It was updated continuously based on new areas found in the transcripts. Themes and sub-themes were cross-checked across the transcripts for consensus. Only major themes are reported in this paper. The topics of pregnancy, postpartum, and newborn care practices were further subdivided into various subthemes, e.g., under the theme of pregnancy, there was diet and rituals followed in pregnancy, phone usage, and support from family during pregnancy and delivery.

## 3. Results

All the 20 recruited women were found to be within the age range of 25–30 years. One participant was illiterate, three had education up to primary level, eleven had up to secondary school graduation, and five had higher than secondary education level. This shows that 95% of our study population was literate, compared to the overall Indian literacy rate of 74.37% and Punjab literacy rate of 75.85% in 2011 (23).

The key findings are presented within three main themes that permeated from a thematic analysis of the present research: (a) Pregnancy practices; (b) Postpartum period practices (c) Newborn care practices. The findings from the qualitative interviews are presented under these three themes.

### 3.1. Pregnancy practices

Different cultures have different values, beliefs, and practices. A woman's cultural background can affect her needs and expectations during pregnancy as well as the practices adopted by her. In our study, many women were influenced by the rituals described or advice given by elders or those close to them, whilst others were much less likely to follow traditional cultural practices. We could see in our study that women follow the dietary advice given by their elders. They were found to follow their family rituals regarding their stay place in pregnancy being their parents' or in-laws' house. They may stay there for a particular time in pregnancy or after delivery depending upon their cultural practice. In this time period, the husband stays at the residence (place of stay) where he was already living and does not move with the pregnant woman.

#### 3.1.1. Diet during pregnancy

In general, women described eating varied and fairly healthy diets in pregnancy. Multiple women followed advice from older generations, such as their mothers or in-laws, on what foods to eat during pregnancy. One woman explains:

*In pregnancy, my mother had told me not to eat anything hot in nature, especially in the beginning. In the initial months, she used to give me healthy things like milk and curd and fruits like pomegranate and apples*.

Women also listened to their elders regarding the food items to be avoided, which included hot foods (garam taseer) or “hard” foods. One woman describes:

*I stopped my intake of hot food items as the elders used to say that hot food items could…harm the baby…I am told not to eat certain vegetables as hard food can cause indigestion…if I am eating apples, the elders will tell me to remove the skin of the apple and then eat. The skin is a bit hard and would not get digested in time*.

#### 3.1.2. Support and mobility in the late pregnancy period

As a part of their ritual practices, whether the delivery location should be the mother's or the mother-in-law's home, the pregnant woman would be sent to her mother's home in the 7th month for a certain amount of time. One woman explains:

*If delivery is to be done there (parent's house), then they (in-laws) send you in the 7th month…they send you with clothes and shagun (money given as a blessing to the child). But, I had to deliver here (in-laws house) that's why I came back after 4–5 days*.

During the last months leading to delivery, women reported generally being provided with extra care from their family members. They were told to eat well and rest more. However, this was not the case for all participants; other women were told that continuing to work and exercise would help them have a normal delivery:

*Everyone used to suggest continuing working for normal delivery. I used to go for a walk in the evening also, as much as possible. This will help in normal delivery*.

One woman followed advice from the elders to engage in religious practices to promote positive effects on the baby.

*I would recite Japji sahib (a prayer) in the morning or would recite Rehras sahib (another prayer) in the evening. I was in the habit of doing so. At times I would play Gurbani (Sikh hymn) whenever I felt like it. It means my whole day would pass on like this. It is said that when there is such a situation (stress, worry) then you should divert your thoughts to something else*.

Going outside of the home was not a common occurrence for the participants. Some women were restricted from going out by family members, while others chose to remain at home as a precaution.”

*When my sister-in-law's baby was born, someone gave her something to eat before delivery, e.g. some sweets; her baby died after delivery. My mother-in-law was a little scared so now she wouldn't allow me to come in front of people; my room is upstairs. I would remain upstairs. I would not go outside; she restricted me from going out. I stayed at home and she or my sister-in-law always stayed with me, they never left me alone at home. It felt special, that was a good time. When mother-in-law is not allowing…. I cannot disobey her and even I was also not interested in going out*.

#### 3.1.3. Using phones during pregnancy

Pregnant women were also advised not to use their phones during pregnancy or after delivery in order to focus on caring for and protecting themselves and their babies.

*After delivery, they used to say to not use the phone for 1.5 months, not to hear anything too noisy… I started watching TV 1.25 months after delivery. It can be harmful to use phones a lot.*

*They used to stop me from watching TV or advise me to use my mobile less because it has an impact on the eyes*.

#### 3.1.4. Support during delivery

Women described certain rules around who could and could not support them during their actual labor and delivery, but overwhelmingly, there was an awareness that women needed some form of social and emotional support at that time. One participant said that a pregnant woman's mother or husband should not be there beside her as they take half of the pain and labor pain gets prolonged. But if the mother-in-law is present, nothing of this sort happens and the pregnant woman faces labor pain easily.

*A pregnant woman's mother or husband should not be there beside her as they take half of the pain and labor pain delays. And if the mother-in-law is present then nothing of this sort happens and the pregnant woman faces labor pain easily*.

*They allowed my sister to come. Actually, it is said that during those 1.25 months, the woman's body is very sensitive and needs extra care. So, my sister took care of me*.

### 3.2. Postpartum period practices

The results show variations in postpartum practices in terms of rituals followed after delivery to celebrate the birth of the baby, how they practice self-care, diet followed after delivery, and practices adopted to move outside or stay inside the home.

#### 3.2.1. Self-care: after delivery

Many acts of self-care after delivery involved physical acts of soothing their own bodies, such as massage, nipple care, and baths.

*They say that there are black-black ‘til (blackheads on the pores of the nipples that need to be removed for the milk to secrete) that do not get removed by themselves; they are to be removed. If not removed, milk will not come…. My sister-in-law told me that you can use a comb also if milk does not come*.

Some respondents acknowledged that they were advised certain things by their elders but they opted not to follow that advice due to their actual circumstances and needs.

*There are rituals but they (elders) said nothing to me; I took a bath with warm water on 2nd day after coming from the hospital. They would say not to sit under the fan but it was very hot at that time so without a fan it wasn't possible, my baby also cannot do without a fan*.

Participants described a number of different cures or remedies for body pain. Some remedies were purely superstitious (keeping a matchstick between finger and thumb), while others contributed to practices related to limiting mobility and isolation during this time period.

*They told me that you have to take care of yourself like…do not put your hands in cold water at all and like in villages, it is said a lot that you should cover your face for 1 month and 15 days, right? Like cover your face as much as possible, and don't expose too much to the air or to the body so that you do not have any problems*.

#### 3.2.2. Mobility after delivery

Women also described restricted mobility in the postpartum period, including restrictions on visiting Hindu temples. This confinement period (40 days) is a time when the mother is believed to be vulnerable and stays indoors/is restricted to the house: “*She cannot go out for 40 days. But she stays with her child”*.

Women described a cultural practice of no one touching them for the first 40 days or taking the child directly from her, as a vaginal birth was perceived to make the woman “dirty.”

#### 3.2.3. Maternal diet after delivery

After birth, many women consumed nutritious warm foods, such as panjiri (whole-wheat flour fried in sugar and ghee), milk, dal (lentils), chapatti, and porridge. Several foods such as panjiri and milk, in particular, were consumed to promote healing and lactation for the mother.

*My mother had made panjiri (high-fat snack), she used to give me milk, she used to ask me to eat warm foods…Mumma gives me milk three times so that I keep producing milk for the baby*.

Other women described not being allowed to eat certain things, especially related to the “temperature” of that food.

Yes, sometimes I crave butter milk but it is cold so I'm not allowed to have it. My husband would say keep it aside,*smiles* he gives me things that are beneficial.

Participants or their families often worried that the foods they were eating could affect their baby's health. As described in the quotes below, this often led to women restricting their diets due to certain beliefs about how it might impact the baby.

*I cherish eating spring rolls but I can’t have them. It is said if you eat fried food, the baby will suffer from a cough…. If somebody gives me something to eat then also, I will think twice before consuming whether it is good for my baby or not. It is better if I don't consume it*.

*It is mostly about my own eating. Like I shouldn’t eat oily things or curd. Because she drinks my milk, she might have some problems. I love curd. I do not eat it because of her. Because baby might get cold if I eat curd, oily things, fast food. During pregnancy, I also never had fast food; they said it is not good for the baby in the tummy. Now I don't eat it because she has to drink my milk*.

In some cases, women were stopped from eating certain foods by their family members, notably mothers-in-law, because those family members had perceptions about the impact of certain foods.

*A few days back, my sister-in-law cooked rice and offered me but my mother-in-law stopped me from eating it as she said it was not good for my health and would directly affect the health of the baby*.

##### 3.2.3.1. Rituals after delivery

Women described a range of experiences of after-delivery rituals to celebrate the birth of their newborn. The rituals often involved intimate gatherings of worship and blessings or large gatherings of families with an abundance of food and gifts. We could observe in the present study that, usually, celebrations or family parties were planned for baby boys.

*After 45 days, somebody throws a party or anything. Mostly it is done for the baby boy only. It is done less for the baby girls. There is just the custom of doing 21 “chwonki” within 45 days…when the girl is 11 or 21 days old…whether only children are invited or do we have to invite the families also relatives. For 45 days…we are not allowed to wear new clothes or go outside…*

However, some respondents described celebrations occurring, regardless of sex: “After coming home from hospital, a lot is done whether it is a boy or a girl; happiness is celebrated.”

### 3.3. Newborn care and feeding practices

Generally, women discussed a few issues with breastfeeding. However, there were some superstitions reported that could cause women to not breastfeed in public:

*They say do not feed the baby in front of some stranger. They say then the baby does not feed. If that happens, then we have to remove the evil eye by holding a thing in the hand, circling it around the baby seven times, and then throwing it in flowing water*.

Respondents described various home remedies used for infant care and illnesses, commonly “gripe water” to help with constipation, massage (often with mustard oil or clarified butter), and rubbing the baby with asafetida. Some respondents discussed having an awareness about not giving any traditional medicine to babies until 6 months as it can cause infections.

Recommendations around the practice of black pencil (kajal, black soot mixed in butter) being commonly applied to the eyes of babies, and some mothers, as described below, caused tension among doctors and family members:

*I cannot apply kajal, I am afraid. Doctors do not recommend applying kajal, as it causes infection, but in the village, as you know, it is said that it will make the eyes bigger. Made by my mother-in-law, I apply that only*.

A number of respondents described giving honey to their babies:

*Yes, sometimes I give him honey. It is good for children you know and it is homemade. After 7–10 days, I give him it and it is good (beneficial) for cough*.

## 4. Discussion

A variety of cultural beliefs impact women's behaviors and care-seeking ability during pregnancy and the postpartum period in northern India. Some of these are impacted by old beliefs and others are more recent, such as those surrounding mobile phone usage.

It has been found in past literature that dietary restrictions are common in pregnancy and childbirth (10). These dietary restrictions and precautions are often observed under the auspice of protecting the health of newborns or promoting women's recovery. Avoiding certain kinds of food at the time of lactation is primarily perceived to be in the best interest of the baby (25). However, optimal food consumption during the postpartum period is crucial to support the additional nutrient requirements for breastfeeding and to reduce postpartum weight (26).

Contrary to cultural beliefs, dietary restrictions deprive postpartum and lactating women of some of the essential nutrients at a time when these are particularly needed: “For example, restrictions on diets, particularly the lack of a substantial meal for the first few days after birth, could have negative impacts on both the woman's recovery and ability to initiate breastfeeding (27–29).”

The quality and quantity of breast milk is also possibly adversely affected by a restricted diet (27–29).

Another common thread in the pregnancy and postpartum period, which is less well described in the literature, is the practice of restrictions on women's movement during the perinatal and postpartum periods. One previous study in India described how a belief that women should not cross a river during pregnancy may restrict women's access to antenatal care (9). In our study, women described limitations on movement, for example, not being allowed to leave the house or visit certain places. Such restrictions could limit their ability to seek care if needed or lead to loneliness, isolation, and potentially postpartum depression (30). Also, in some Indian Hindu families, the whole family is considered impure during the postpartum period of the mother. No outsiders are allowed to eat or drink in the house until a day determined by caste and until a ritual bath and religious ceremony are performed (31).

However, it was interesting to find that some women described that being restricted to the home felt special, and it seemed to be viewed as a marker of being cared for. Thus, this practice can potentially, at least in certain circumstances, be positive for some during this period. Relatedly, overall, the women in our sample seemed to feel supported during pregnancy and the postpartum period, which is likely to lead to better pregnancy and postpartum health outcomes (32, 33).

There is also a practice of restrictions on women's movement during the perinatal and postpartum periods. The various reasons for these are the norms around privacy for breastfeeding, which could potentially reduce breastfeeding exclusivity or duration, especially if women have to work outside the home and are unable to breastfeed in public.

In India, different ceremonies may or may not be practiced after birth based on the sex of the baby. There is a great deal of literature on gender preferences, where some women see having a son as a greater blessing and express disappointment in having a daughter (12, 14). While holding a ceremony may not in itself be harmful to the infant's or mother's health, these practices internalize and perpetuate gender inequality and could also cause emotional distress and depression to mothers who do not produce a male child. This is a reflection of gender inequality and unequal gender norms. It is important to note that nowadays, many families celebrate the birth of a girl, which may be impacted by social media, but this was not supported by the study population. Only a few respondents were of the opinion that a lot is done whether it is a boy or a girl; happiness is celebrated.

Another finding was related to restrictions on women's phone usage while pregnant and during the postpartum period due to beliefs about the potential ill-health effects of exposure to phones on women and infants. It is important to consider this in view of the low care seeking from health facilities by the family accompanied by less-than-optimal home visitation by the providers. This can seriously compromise the health of the mother and the baby, despite mhealth platforms providing an increased possibility of reaching women with accurate health information and support (33).

There is a recent and growing body of literature from India describing the intricacies of the gender gap in phone usage. A recent paper outlined how, even if, nominally, women have access to a phone, its use is very restricted due to gender norms around usage, including who gives permission for the phone to be used and for what purposes it can be used (34).

A few infant practices were described which are causes for concern, particularly the practice of feeding babies honey and of putting kajal (black soot mixed in butter) on the eyes, which has also been found in previous studies (10, 12). Honey, or any processed foods containing honey, should not be given to children under 1 year of age because introducing honey too soon may cause infant botulism by Clostridium botulinum spores found in honey and honey products. These spores turn into bacteria in the bowels and produce harmful neurotoxins in the body (35).

Kohl (surma) is an eye preparation in the ultra-fine form of specially processed “Kohl Stone” (galena) mixed with other active ingredients (36).^.^ It has been found to be used in babies with the idea of keeping the eyes cool, improving vision, strengthening the eyes, and protecting the baby from the evil eye. Studies have revealed that “Kajal” comprises galena (PbS), minium (Pb3O4), amorphous carbon, magnetite (Fe3O4), and zincite (ZnO) (37). Prolonged application may cause excessive lead storage in the body, affecting the brain and bone marrow, and causing convulsions and anemia (37). Dirty fingers and sharp and uneven fingernails of the caregivers are potentially harmful to the child's eyes (37). There is a need to concentrate on health education regarding the potential harm of some of these practices in the communities where such customs are followed.

## 5. Conclusion

Understanding community practices and beliefs is a vital step in improving early neonatal outcomes, because only after identifying knowledge gaps and harmful behavior can community-based programs be tailored to the needs. Our findings have identified several socio-cultural practices that need to be addressed to help improve maternal and infant health outcomes and prevent unintentional harm. Many traditional practices remain in the pregnancy and postpartum period in India, but it appears that new behaviors and beliefs are also arising, which are at the intersection between culture (gender norms) and technology.

This study has many strengths, including looking in depth at an understudied set of practices through qualitative interviews with women living in rural communities in northern India. However, as with all studies, there are limitations. The present study was conducted using a small sample size; with study participants from only one rural area. Expanding the project into other locations would be beneficial. Additionally, we only collected data from women, and since decisions are often made at a household level, collecting the opinions and views of other family members would enhance future research.

Developing strategies that address and, where possible, integrate mothers' traditional beliefs and modern approaches to postpartum care are essential. Additionally, developing culturally sensitive messaging to help reduce potentially harmful practices and strengthen positive ones (such as social support) should also be a priority among programs and policymakers. Despite declining maternal and neonatal mortality, it remains important to focus on the promotion of an exclusive breastfeeding diet and adequate fluid and nutrition for the mother. Further research exploring other cultural practices (e.g., religion/spirituality) in this location and practices in other locations would be valuable.

## Data Availability

The data that support the findings of this study are available from the corresponding author upon reasonable request.
